# Something about Eyes

**Published:** 1887-09-03

**Authors:** 


### Till the Doctor Comes.

Inquiries and Communications for this column should be addressed "Physician," care of the Editor of The Hospital.]

### XXV.—SOMETHING ABOUT EYES.

*Squint.*—This, in ninety-nine cases out of a hundred, is a sure indication that the child requires glasses. Of course a surgical operation is sometimes necessary. The tales as to the origin of a squint from mimicking a squinting person are generally apocryphal. A squint should always be attended to, as, if long developed, the child learns to see with one eye only, and the sight of the affected eye becomes much impaired, and may even be lost.

*Spectacles.*—These should always be chosen under the advice of a skilled oculist, not haphazard. They are usually made of crown or flint glass. Some are made of rock-crystal, when they are known as pebbles, but they are not superior to the glass. When spectacles are used only to protect the eye from the glare, probably neutral grey are the best. Blue glasses and glasses of any colour, if long used, render the retina unduly sensitive to those colours which are excluded. The spectacle-frame should be made to fit correctly, so that the centres of the glasses may be exactly opposite the pupil.

*General Rules.*—It should be remembered that in distant vision only is the eye at rest, and if prolonged close work must be continuously performed, the eyes should be occasionally rested by withdrawing them from the work for a few moments. Reading in a train or carriage is bad, as the constant jarring alters every few seconds the relative positions of the book and the eye, causing continued action of the ciliary muscle and consequent fatigue. The reading of very small print should always be avoided under these circumstances. Reading when lying down is also unadvisable, as it is almost impossible to place the book in a favourable position, and the muscles of the eyeball are strained. Attention should be paid to the type of a book. The size of the letters, the good and distinct quality of the type and shade of the paper are each of them important. Gaslight is not a good light for the eyes, as it contains so many yellow rays. Some of the oil-lamps are best for close work at night. They require shades, however, as most of them are of such a height that otherwise their full glare falls upon the eye. This is scarcely the place to discuss diseases of the eye; but attention may be drawn to one or two small practical points—(1) Never neglect the inflammation of the eye which so often occurs in new-born infants, and is attended with a great deal of discharge. Such neglect is a most fertile cause of blindness, especially among the poor; and if taken at once the disease is generally cured without any difficulty. (2) Recollect that a foreign body in the eye (as sand, grit, etc.) causes almost exactly the same symptoms as a "cold," but the evil will not be cured until the foreign body is removed. (3) A cold in the eye simulates, to an unprofessional gaze, numerous more serious diseases. If it does not soon yield to home treatment, skilled advice should be sought.

(*To be continued.*)

